# A Psychosocial Support Program for Young Adult Childhood Cancer Survivors in Austria: a Qualitative Evaluation Study

**DOI:** 10.1007/s13187-021-02083-2

**Published:** 2021-09-14

**Authors:** Thomas Pletschko, Kerstin Krottendorfer, Juliana Schlifelner, Agathe Schwarzinger, Verena Fohn-Erhold, Liesa Weiler-Wichtl, Anita Kienesberger, Ulrike Leiss

**Affiliations:** 1 Department of Pediatrics and Adolescent Medicine, Medical University of Vienna, Vienna, Austria; 2 Austrian Childhood Cancer Organization, Vienna, Austria

**Keywords:** Childhood cancer, Young adult survivors, Psychosocial support, Aftercare

## Abstract

Many adolescents and young adult (AYAs) childhood cancer survivors face disease- or therapy-related late-effects, which limit their participation in various areas of daily life. AYAs are often left alone in our health care system, and many worry about their ability to cope with long-term sequelae, and some are even lost to follow-up. Therefore, in the present study, a targeted aftercare program was developed and evaluated with the goal of facilitating three important “life skills”: (1) self-perception, (2) social interaction and conflict management, and (3) self-conscious communication of support needs. A total of *n* = 13 participants (19.2–30.2 years, mean age 22.8 years) completed a 3-day aftercare seminar, at the end of which each participant wrote a reflection letter (“letter to my future self”), elaborating on observed effects of the seminar, applicability of the given information in daily life, and the direct impact of the seminar on their individual circumstances. The reflection letters were analyzed using qualitative content analysis. All target life skills were mentioned in the reflection letters. The participants reported individual benefits from the program especially with respect to self-perception and self-confidence, giving and taking feedback, and acceptance of personal strengths and weaknesses. Moreover, the feeling of “not being alone” was associated with the survivors’ experience of emotional and social support. This evaluation highlights the potential of a one weekend aftercare seminar to address important life skills that are known to positively influence health behavior in AYAs. The detailed description of the seminar can serve as a basis for making this kind of aftercare accessible for other people in similar circumstances.

## Introduction

Adolescent and young adult (AYA) oncology patients (ages 15–39) have been identified as a group with healthcare disparities [1]. Owed to medical progress, the chances of recovery following successful cancer treatment have improved substantially in the last decades [2]. Thus, for AYAs, the quality of life and the disease- or therapy-related late-effects have gained substantial importance in the medical and psychosocial context [3]. In fact, AYAs face a variety of disease- or therapy-related challenges such as handling disabilities and coping with visible signs of the illness [4, 5]. Many of these late-effects only appear years after therapy and which makes coping an ongoing process [6]. Simultaneously, AYAs have to master the whole range of normative developmental tasks (i.e., tasks that are common to all individuals of this specific age group, such as building relationships, starting a career, living independently) that are typical for the developmental stage of “emerging adulthood,” which is defined as a prolonged period of independent role exploration during the late teens and twenties [7]. Accordingly, in their qualitative study, Patterson et al. [8] investigated the needs of emerging adults with a cancer diagnosis and identified four main requirements: accepting responsibility for oneself, deciding on personal beliefs and values, establishing relationships with parents as equals, and becoming financially independent. Furthermore, numerous studies indicate that the consequences of not achieving the developmental tasks can lead to unemployment and social isolation [9–12]. Hence, the development of young patients’ personal identities can be considered a crucial task in the transition from adolescence to young adulthood. Thereby, long-term medical and psychosocial aftercare programs can be helpful in promoting these essential skills [13–16].

Research focusing on the effects of cancer showed that many patients, particularly those with a neuro-oncological cancer history [17–19], are at a high risk for long-term physical, social, emotional, and cognitive consequences following illness or treatment [20–26]. These findings underline the importance of sophisticated aftercare programs for long-term survivors, and interventions aimed at promoting the attainment of developmental tasks, such as training of advocacy skills [27], are generally recommended [28–30]. With respect to medical aftercare, structured long-term follow-up systems and guidelines for long-term follow-up for adult survivors of childhood cancer are already being developed across Europe and North America [31]. To address the specific population of AYAs, manuals dealing with long-term survivorship and the transition from adolescence to adulthood have been established in different parts of the world over the past years [32]. These guidelines are highly useful in providing treatment guidance and informing professionals about the specific needs of young patients. With respect to psychosocial follow-up, only two guidelines could be identified: (1) The guidelines of the professional society PSAPOH (Psychosoziale Arbeitsgemeinschaft in der Pädiatrischen Onkologie und Hämatologie—Psychosocial Association in Paediatric Oncology and Haematology) [5], which emphasize the necessity of aftercare as a phase-specific psychosocial intervention, and (2) the comprehensive standard of care model in pediatric psycho-oncology, published by the SIOP-PPO Organization (International Society of Pediatric Oncology – Pediatric Psycho-Oncology Group) in 2015 [33]. Both the PSAPOH and the SIOP-PPO guidelines highlight the need for an interdisciplinary network of follow-up institutions as well as the preparation of transition programs to structure the shift from pediatrics to adult medicine. However, evidence-based guidelines for psychosocial aftercare of young adult survivors of pediatric cancer are still lacking.

The aim of the present study was therefore to assist the participating AYAs in attaining normative developmental tasks by promoting the improvement of their life skills necessary for a structured transition into adulthood. Hereby, the term “life skills” refers to the ability of exhibiting adaptive and positive behaviors that enable individuals to effectively deal with the demands and challenges of everyday life [34]. These skills include decision making, effective communication, interpersonal relationship skills, and self-awareness, among others. The goal of the program was to foster a positive perspective of the AYAs on their own health and personal character traits, and to provide them with guidance to grow into autonomous individuals within society.

In a recruitment setting, important skills are typically evaluated by means of an assessment center (AC), which is defined as an investigation technique, based mainly on behavioral observations. Furthermore, ACs can be used as an instrument for developing human resources and capacities [35]. In this study, three AC techniques were adopted and modified for assessing and promoting relevant life-skills in AYA Childhood cancer survivors. Taking the findings of recent literature on the promotion of life skills [27–29], and the Austrian Childhood and Youth Health Report [36], three basic life skills could be derived: (1) self-perception, (2) social interaction and conflict management, and (3) self-conscious communication of support needs. A detailed explanation of these concepts can be found in Table 1. The present study evaluates these three abilities using AC-behavioral simulations.

**Table 1:** Definition and description of the basic life skills that are trained in this program

Self-perception	Social interaction and conflict management	Self-conscious communication of support needs
- … allows individuals to find out about own emotions based on the observation of their own behavior [28]. Because of internal cues that are too weak, ambiguous or too difficult to interpret, external observable cues can be used to draw conclusions about own internal states [28, 29] - … is associated with the ability to gain an image of ourselves (self-reflection [37])’, i.e. individuals explore and analyze their behavior in past situations; thus, new insights and perspectives can be gained [38, 54], which, in turn, can be transferred to current and future life situations [38]). This process is an important learning experience; whereby behavioral changes can be initiated [38] - … contributes to personal growth through self-awareness [39]. This explains why self-reflection is of great importance in various areas of life (e.g. work, education) [38]	Social interaction: - is an interdependent process, in which messages are exchanged between interaction partners [40, 41]. This communication can be considered as an important basis for all kinds of social interaction [42]. Communication itself can be seen as a social process in which the participants interactively construct a reality [43]. However, wherever people meet and interact with each other, conflicts may arise Conflict management: - Conflicts can be referred to as incompatible tendencies of action [44], which represent a natural process within social interaction [45]. Opposing opinions, attitudes, or behaviors are ubiquitous and normal. Only if the experience is disturbing, conflict-management is necessary [46]	- Communicating needs and actively asking for help are important self-regulatory strategies. It can positively contribute to students learning [47] and to problem solving and learning in organizations [48]. Regarding cancer survivors Zebrack et al. [49] showed that attendees of the advocacy skills training for young adult cancer survivors reported they felt more confident about themselves and how they wanted to direct their lives after this training - These findings suggest that survivors benefit from the ability of communicating their own support needs in a self-conscious way - The prerequisite for this is being aware of one’s own strengths and weaknesses, individual characteristics and belonging to social groups, what can be summed up as the self-concept [50, 51]. Survivors should therefore be trained in having an adequate self-concept that enables them to know where they need support and how to communicate this

One of the most important principles of ACs is feedback, which is defined as a particular evaluative form of communication [52] in response to interpersonal processes and preceding actions [53]. Feedback can be active or passive — active feedback occurs when the sender expresses praise, criticism, or advice directly to the recipient; passive feedback is obtained by observing other people and drawing conclusions about one’s own behavior based on the observations [52]. Implementing received feedback is significantly linked to self-reflection [54] and contributes to the construction of an external image, which can in turn be integrated in the self-image. A reciprocal process of giving and taking feedback can significantly facilitate and improve interpersonal relationships in various social aspects of our life [52, 53]. Therefore, feedback is particularly important in terms of social interaction and conflict management.

Taken together, the aim of this study was to develop, conduct, and evaluate a targeted intervention program for AYAs, focusing on the above-mentioned life skills, in order to promote the attainment of developmental goals, by means of using assessment center-based behavioral simulations and feedback techniques. The main research question was whether the newly developed program can provide AYAs with an individual benefit for everyday life challenges.

## Methods

The 3-day-seminar consisted of a psychosocial aftercare-project from the Austrian Childhood Cancer Organization. In accordance with the theme “Offer strength and hope—Survive!,” the Childhood Cancer Organization has been organizing camps for children and teenagers suffering from cancer for over 20 years to help them regain control of their lives, deal with their fears, and improve their self-confidence [55].

### Participants

The target group of this intervention are AYA cancer survivors aged approximately 20 to 35 years (for younger AYAs, aged 15 to approximately 20 years, a separate program exists), who believe that cancer- or treatment-related late-effects have a negative impact on their lives. Information about the seminar was distributed via email and social media channels of the Austrian Childhood Cancer Organization as well as directly by psychosocial staff of aftercare units in various Austrian hospitals. Participants were asked to register by phone or email via the Austrian Childhood Cancer Initiative’s office.

A total of 14 AYAs attended the seminar, although one had to leave one day earlier because of acute fever. Therefore, a total of 13 participants (*n* = 13) completed the seminar and took part in the evaluation. There were 9 female (69.2%) and 4 male (30.8%) participants. The average age was 22.8 years (range 19.2–30.2 years). Twelve AYAs suffered from a neuro-oncological disease in childhood or adolescence (93%, 5 Medulloblastoma, 2 Ependymoma, 2 Pilocytic Astrocytoma, 1 Craniopharyngeoma, 1 PNET, 1 Pineoblastoma), and one participant had leukemia (7%). The mean age at diagnosis was 7 years (range 2.2–12.5 years), and the mean time since diagnosis was 15 years (range 8–26.5 years). Of the 13 participants, 11 had received chemotherapy, 9 radiotherapy, and 12 had a neurosurgery.

### Development of Seminar Concept

The seminar was conceptualized as “assessment-center-weekend” for the AYAs; hence, the participants were divided into two groups: one group took part in the behavioral simulations (performers); the other group acted as assessors. Performers pretended to be job applicants; assessors assessed their competence on the respective task. The roles were exchanged afterwards. The group constellation changed for every exercise. At the beginning, the participants were trained for describing human traits and abilities by teaching them well-established observational and feedback-techniques. Based on these ideas, the participants developed a monitoring system in the form of an observational sheet which was used to evaluate various situational exercises such as group discussions, role plays, or self-presentations (e.g., in one role play, participants were confronted with individual weaknesses by their boss; they had to explain their concerns and ask for support; the addressed weaknesses were typical for real life situation of AYAs). After each exercise, AYAs in the assessor group provided feedback to their peers based on their observations, whereby the sandwich-feedback-method was applied. In addition, all participants received verbal feedback from seminar facilitators for each exercise and written feedback at the end of the seminar. The aim of all exercises was to promote the target abilities, namely, (1) self-perception, (2) social interaction and conflict management, and (3) self-conscious communication of support needs. The concept of the seminar is compiled in Table 2.

**Table 2:** Modules of the seminar

Time period	*Module*	*Target figure*
*Friday evening*	Joint dinner	- First acquaintance
	Introduction to the topic	- Icebreaker “common properties”
		- Brainstorming: assessment of human traits^a^
		- Development of a monitoring system
*Saturday morning*	Self-presentation “myself as survivor”	- Communication about own strengths and weaknesses
		- Self-assessment
		- Giving and receiving feedback
*Saturday afternoon*	Group discussion: (a) Neutral topic: “NASA-game” (b) Emotional topic: “Dealing with late effects”	- Group communication
		- Reflectivity
		- Giving and receiving feedback
*Sunday morning*	Role-playing — application situation: explaining cancer-related late effects^c^	- Communication about own strengths and weaknesses
		- Give and take of feedback
		- Reflectivity
		- Self-assessment
*Sunday noon*	Conclusion	- Evaluation — letter to myself
		- Reflectivity
		- Outlook – future issues
*Additional*	Brain games	- Neurocognitive strengths and weaknesses^b^

^a^For example, communications skills, conflict ability, cooperation capability, problem-solving ability, and decision-making ability

^b^For example, memory, concentration, processing speed, planning and organization, flexibility, and inhibition

^c^Remark on evaluation: because of lack of time, it was not possible to perform the module role-playing

The seminar was designed to be applied by a clinical psychologist and a social worker as seminar facilitators, whom should be assisted by one or two attendants depending on the number of participants. The professionals should have a background in childhood oncology of at least 3 years to guarantee their ability to identify the special needs of survivors.

The seminar is designed to be residential and hence meant to take place in a hotel or AYA-aftercare facility with a duration of three days and two nights. The focus of the seminar was the confrontation of AYAs with their personal character traits, to identify their own strengths and weaknesses and to encourage a self-reflective process as recommended by the previous studies. In this context, weaknesses are understood as cancer- or treatment-related long-term effects.

### Evaluation Design

The present study aimed to assess the survivors’ subjective benefits drawn from the intervention with respect to personal development. Furthermore, the participants’ ability to transfer the improved or newly acquired skills from seminar to everyday life was evaluated. Since transfer effects were not assessed directly, the results refer to perceived possibilities.

Data collection for the evaluation was part of the seminar and therefore conducted by the seminar facilitators (one clinical psychologist and one social worker). Afterwards, the project was evaluated by two psychologists who were not involved in the development of the seminar concept. To answer the research question of what individual benefits the program could offer to the person’s daily life, the participants were asked to write a reflection letter (“letter to my future self”) at the end of the seminar, following a specific guideline. This guideline contained questions on the observed effects of the seminar, applicability of the provided information in everyday life and the direct impact of the seminar on the participants’ individual circumstances.

The method of analysis followed the qualitative content analysis according to Mayring [56]. The aim was to systematically filter the relevant aspects to devise a system of categories from the text materials [56]. This work was done by a psychologist, who was not involved in the development of the program and who acted as head of evaluation. The inductive analysis comprised five steps: (1) transcribing, (2) paraphrasing, (3) generalization, (4) first reduction/removal of identical contents, and (5) second reduction, summarizing and categorization [56]. The second psychologist was included in the coding process as a second rater. There were no major modifications, which demonstrate the good quality of the coding system.

## Results

### Qualitative Content Analysis

A total of 13 participants wrote the “letter to the future self.” The analysis was built around the following predefined topics: “observed effects of the seminar,” “applicability of the given information in daily life,” and “impact on the individual circumstances.” These topics comprise several categories, which in turn constitute the main contents of the reflection letters (for an overview see Table 3).

**Table 3:** Categories and generalized paraphrases

*Topics*	*Categories*
Observed effects of the seminar	(1) Self-reflection
	(2) Acceptance
	(3) Strengths and weaknesses
	(4) Feedback
	(5) Self-confidence
	(6) Emotional and social support
Applicability of the given information in daily life	(1) Self-assured manner
	(2) To be proactive
	(3) Self-reflection
Impact on the individual circumstances	(1) Self-presentation
	(2) Social skills
	(3) Self-acceptance
	(4) Autonomy

### Observed Effects of the Seminar

1. Self-reflection: The participants received support for their self-reflection as they learned to *identify themselves with their own personalities* and to *perceive, satisfy, and acknowledge their personal needs*. A crucial aspect here is the *reflection and recognition of personal strengths and weaknesses*. For example, participant 5 wrote that she/he had learned “to become aware of her/his own strengths.” Related to the reflection of their own lives and medical history, the participants learned to better *verbalize their stories*.
2. Acceptance: This aspect includes *accepting their own life situations*, more specifically, accepting long-term changes and life conditions, as well as one’s own strengths and weaknesses. Those experiences could also contribute to the *development of a positive attitude*.
3. Strengths and weaknesses: Two central aspects of learning in this category include the *reflection and handling of personal strengths and weaknesses*.
4. Feedback: The participants could gain experience in *giving and receiving feedback*.
5. Self-confidence: The participants increased their self-confidence by learning to act *self-assuredly*. An important aspect of learning in this category is not to be superficial and *to never judge a book by its cover*. Person 2 wrote: “I realized that it is not about what you look like, but about inner values.” Another important aspect is learning about one’s right to *express opinions* and *to be open and honest* with everyone.
6. Emotional and social support: Participants understand that it is a precious enrichment to know that “others are just like me” (person 8) and to *gain confidence about not being alone with their medical history and problems*. Furthermore, the feeling of being *completely accepted* promotes self-confidence and is hence a very important resource. In this context, person 10 wrote that she/he had also learned how important *collaborative skills* were. This aspect includes engaging with others, supporting them, and, in return, accepting their support.

### Applicability of the Given Information in Daily Life

1. Self-assured behavior*:* The participants stated that various aspects from the seminar helped them with acting more self-assuredly, by *presenting their own opinions, wishes, and needs* and by *showing the newly gained strengths*, which is in turn beneficial for their self-assessment skills. In this context, person 5 wrote that she/he can use the content of the seminar for applying her own strengths better, as well as searching for situations in which she can show and use them. Additionally, the feeling of *not being alone with the medical history and problems* provided the AYAs with more confidence and safety.
2. To be proactive: The respondents stated that the new experience had helped them to be more active in their daily lives. This includes aspects such as the *implementation of new things from the seminar in their daily routine*, e.g., *giving feedback*. Moreover, the learning aspects can contribute to *coping more easily with their after-effects* and problems in their everyday lives. This is a substantial part of being proactive when it comes to living an independent and self-determined life.
3. Self-reflection: The young adult survivors listed that the learned content had helped them to be more self-reflective in their routines. The most beneficial content for their daily lives was thereby *perceiving and accepting their personal needs* which the participants find helpful in *handling their own thoughts and feelings*.

### Impact on Their Individual Circumstances

1. Self-presentation: Various aspects from the seminar were described to facilitate the ability to *exert confidence* and *show personal strength*.
2. Social skills: The respondents stated that the learned content had helped them to *integrate more easily into society*. The important increase in social skills includes the ability to *assert oneself over others*, to *hold conversations* and to *give and take feedback*.
3. Self-acceptance: This category includes self-acceptance concerning personal strengths and weaknesses for coping better with one’s *own feelings and emotions*. In this respect, person 11 wrote that the newly acquired knowledge helped her/him “to not always see things negatively because the grass is not greener on the other side, and everyone has problems to cope with.” In a broader sense, this skill could contribute to *gaining a positive attitude* as well as *living a fulfilled life*.
4. Autonomy: The last avail identified by the participants was the promotion of independence. Various young adult survivors stated that they could benefit from the seminar with regard to *vocational and future aspects* and in this way *gain independence and individual responsibility*.

## Discussion

Overall, the participants perceived the seminar as beneficial and each participant observed an improvement of individual strengths, special experiences, or support. In line with the goals of the seminar, the reflections included all intended aspects and even additional experiences were reported, such as strengthening one`s own self-assurance, learning self-acceptance, and experiencing social support. It is important to note that the changes discussed below are perceived changes, not actual changes, since the evaluation method in this study was a subjective letter written to the future self of the participants. Nevertheless, the subjective impression is an important one in the development of the life skills.

With respect to the first of the three target life skills of this study, self-perception, prior research has shown that many long-term survivors have to cope with disease or therapy-related long-term effects. Particularly changes in patients’ appearance (e.g., scars, sparse hair) create a burden, which can affect various life domains (e.g., social participation) [57]. Hence, accepting one’s own life situations and increasing self-confidence are crucial learning aspects for AYA cancer survivors. The results of this study suggest that via the program the participants learned to better identify themselves with their own character traits and their personal needs. They were required to reflect on their own strengths and weaknesses and reported the impression that they learned to verbalize their stories better. In addition, the participants perceived a process of accepting long-term changes during the seminar and were able to develop a more positive attitude towards their own life conditions. The respondents stated that they anticipate a manifestation of these benefits throughout their daily routine. For example, the aspects of self-acceptance contributed to living a fulfilled life and improved the ability to cope with their own feelings and emotions. This can be seen as enrichment, due to the high risk for anxiety, depression, or problems related to regulation of emotions in childhood cancer survivors [58].

Regarding the second target life skill, namely, social interaction and conflict management, the evaluation letters showed that participants experienced emotional and social support during the residential seminar, including a sense of social belonging as well as the assurance that they are not alone with their medical history and the associated problems. This is an essential lesson to be learned, since social support is one of the main coping-strategies necessary for returning to normal life as fast as possible [59]. Increased self-confidence, social competences, and independence are particularly relevant in terms of participation in the social, school, or professional context. Studies have shown that social participation (e.g., work, financing, daily social functioning) of longtime survivors is often impaired in the long run [8], which can in turn impede self-confidence [60]. The results of this study suggest that the intervention could successfully use social participation as a means of improving subjective self-confidence. Furthermore, the participants reported gained experience in giving and receiving feedback, which is considered an important basis for conflict management. Peer feedback seems to have an especially large impact on behavioral change [61]. However, when considering the definition of conflict management (cf. Table 1), this life skill contains more elements not addressed by the participants in their evaluation letter. A specific training in conflict management could therefore be a future improvement of the support program.

Finally, with respect to the third target life skill: *self-conscious communication of support needs*, participants reported an increase in self-confidence and in self-assured actions. During the weekend-seminar, the AYAs perceived an improvement in expressing their own opinions and being open and honest with everyone. Furthermore, the participants considered the knowledge that other people have had similar experiences to be especially enriching, since it fostered the feeling of being completely accepted by others and led to a behavior where reciprocal support was given and accepted. In addition, a high degree of social support has a positive effect on self-efficacy and well-being [62, 63].

In conclusion, the results support the idea that AYAs childhood cancer survivors, on average 15 years after diagnosis, can benefit from a support program which facilitates the development and improvement of important life skills. Thereby the exchange with like-minded people appeared to be an especially important resource of support regarding the participants’ self-perceptions, which is in line with findings, that patient to patient interchange is rated even more valuable than the support from family and friends, as well as experts and professionals [64, 65]. Since psychosocial after care following medical therapy is often described as necessary for the personal development and social establishment of childhood cancer survivors [66],

### Study Limitations

In summary, the results of the present study indicate that by means of a 3-day weekend seminar, it is possible to enhance self-perception and to stimulate social interaction and conflict management. Furthermore, our results show that self-conscious communication of support needs could be observed and accepted as personal support. However, certain limitations must be acknowledged.

Despite the high motivation and the time used for writing the letters to the future self, some letters were very short, in abbreviated form, with simple formulation, offering only a limited amount of data. From this, it appears that some participants had difficulties with age-appropriate writing skills/expressions.

A quantitative analysis about the frequencies of the new learning aspects and the benefit for daily life, as often claimed [56], was not possible based on this limited material. In addition, the attendance of the seminar was open for all survivors in Austria although the registration had to be done by the participants themselves. Due to the process of self-application, this program might have attracted already highly motivated and more open-minded young adult cancer survivors with a greater ability to accept social support. Another hypothesis is that mainly AYAs with a higher degree of late effects were attracted by the program or it was promoted more among this special group, as all but one participant had a neuro-oncologic history. Nevertheless, the program was not constructed specifically for a group of patients with CNS-tumors and it is conceivable that patients with other cancer diagnoses might benefit from such a support as well.

Finally, data was drawn from individual letters, indicating the personal opinion of AYAs at a certain point in time; evaluation did not include follow-up assessments to judge long-term effects of the program.

### Future Directions and Clinical Implications

This aftercare program can be a model for further offerings for AYAs. To work efficiently on the target domains and to enable long-term effects, from a clinical point of view (as well as from feedback of survivors and their parents), seminars for survivors should be offered at least twice a year (which is the way it is established in Austria). Important skills were trained suited to each participant`s needs. A focus on the transition to their daily lives should be emphasized in future seminars. As illustrated, participants of the seminar gained several competences applicable to their daily routines (at least in their subjective perception immediately after the seminar). However, singular events like this seminar have limitations regarding their range of influence. Consequently, future research is necessary to develop further seminars and treatment programs to address further topics and needs of AYAs and to evaluate long-term effects of such seminars.
